# Seeing in the dark: High-order visual functions under scotopic conditions

**DOI:** 10.1016/j.isci.2024.108929

**Published:** 2024-01-17

**Authors:** Ayelet McKyton, Deena Elul, Netta Levin

**Affiliations:** 1 fMRI Unit, Department of Neurology, Hadassah Medical Organization and Faculty of Medicine, The Hebrew University of Jerusalem, Jerusalem, Israel; 2 Edmond and Lily Safra Center for Brain Sciences (ELSC), The Hebrew University of Jerusalem, Jerusalem, Israel

**Keywords:** Natural sciences, Biological sciences, Neuroscience, Systems neuroscience, Sensory neuroscience, Cognitive neuroscience

## Abstract

It is unknown how and to what degree people function visually in almost complete darkness, where only rod photoreceptors are active (scotopic conditions). To explore this, we first tested scotopic acuity and crowding. We demonstrated the ∼1° foveal scotoma and found that crowding increases with eccentricity, resulting in optimal scotopic discrimination 2° into the periphery. We then investigated whether these limitations affect high-level foveal tasks. We recorded eye movements while testing reading and upright/inverted face matching under photopic and scotopic conditions. Under scotopic conditions, participants read accurately and showed a face inversion effect. Temporally, fixation durations were longer. Spatially, surprisingly, participants did not avert their gaze 2° into the periphery. Instead, they fixated on similar locations as under photopic conditions, locations that were shown to correlate with global perception. We propose that this result suggests global perception governs under scotopic conditions, and we discuss how receptive-field properties support this conclusion.

## Highlights

• High-level vision under scotopic (dark) conditions is assessed for the first time
• Surprisingly, people read accurately and show face inversion effect even in the dark
• People fixate at similar positions in a sentence or on a face as they do in the light
• Results suggest global mechanisms are unharmed and govern under scotopic conditions

## Graphical abstract

**Figure:**
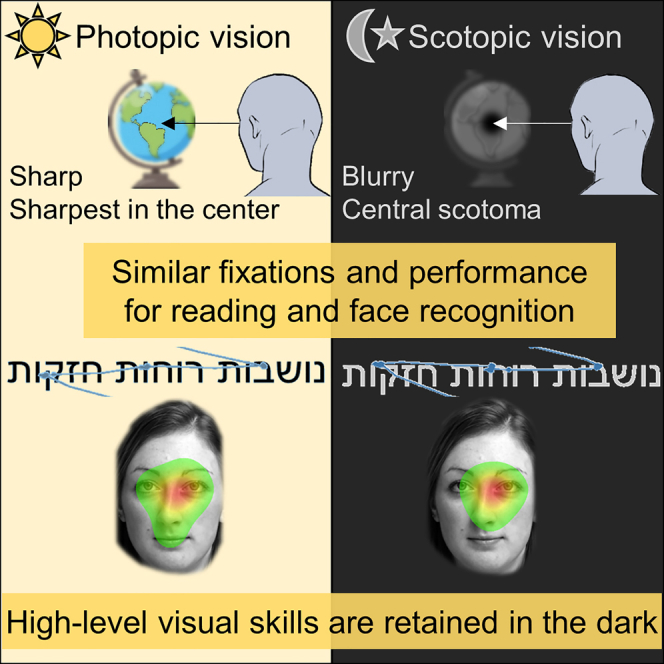

## Introduction

Vision is considered our most valued sense,^1^ allowing us to connect with our surroundings, perform numerous tasks, and stay safe. It is therefore not surprising that high-level visual tasks are constantly being researched to deepen our understanding of visual behavior. However, until now, this research has been almost completely restricted to daylight conditions, neglecting the fact that our ancestors lived in near darkness for half their lives.^2^ The need to visually function under near-dark conditions is probably the reason that rods, the photoreceptors that function in lower light, comprise a remarkable 95% of our retinal photoreceptors.

Scotopic vision, which occurs in near-dark conditions where only rods are functioning, has its limitations. First, unlike cone photoreceptors, which come in three spectral classes which are used to differentiate wavelengths, rods have only one type, causing rod-only vision to be colorless.^3^ Second, spatially, rods are completely absent from the foveola (where cones are most concentrated), resulting in around 1° of foveal scotoma under scotopic conditions. In the periphery, scotopic acuity remains low^4^ due to extensive pooling of the rod signals by peripheral retinal ganglion cells.^5^ Third, temporally, while cones respond at a rate as fast as 100 Hz,^6^ rods are at least 5 times slower.^7^^,^^8^ In summary, scotopic vision is slow and colorless, with a scotoma in the fovea and low acuity in the periphery.

Despite these limitations, humans can still perform numerous high-level visual tasks under scotopic conditions. Nevertheless, only a few studies have tested a complex visual behavior under such conditions (reading,^9^ global form and motion,^10^^,^^11^^,^^12^ stereopsis,^13^^,^^14^ directionalization,^15^^,^^16^ and visual search^17^). The latter study, which was the only one to evaluate eye movements, showed that under scotopic (compared to photopic) conditions, subjects executed longer and less widely distributed fixations when performing visual search tasks.

Taking the foveal scotoma limitation into consideration, one might expect that under scotopic conditions, people would avert their gaze away from an object for better recognition. It has been shown that in the case of a 4° artificial central scotoma, healthy participants shift their eye position upward.^18^ Similarly, in cases of age-related macular degeneration (AMD), researchers found that when a 10–20° fovea is no longer functional, most patients develop an eccentric preferred retinal locus for fixation.^19^ However, this shift in fixation can take days (in the artificial scotoma case), months, or even years (in the case of AMD) to emerge. The question remains whether in the case of a scotopic foveal scotoma, which is significantly smaller and appears occasionally and naturally over the course of our lives, a similar behavior would be detected.

We set out to investigate the nature of the eye movement patterns under scotopic conditions in high-level foveal tasks, specifically reading and face recognition, that usually demonstrate specific and characteristic eye-movement behavior under photopic conditions.

When testing reading performance, it is also important to consider the crowding effect, a major bottleneck for visual object identification which makes it harder to identify a target (e.g., letter) in proximity to other targets.^20^ Under photopic conditions, the crowding interference effect increases with target eccentricity^21^^,^^22^ and severely limits the ability to recognize objects, mostly in the periphery. It is therefore crucial to measure scotopic crowding along the eccentricity axis to understand the effective scotopic acuity for reading.

Under photopic conditions, reading involves specific and characteristic eye movement patterns. Skilled readers make fixations lasting about 200 ms, with a mean saccade size of 8 letter spaces.^23^ They rely on the external features or the word’s envelope,^24^ recognizing the word holistically. Skilled readers’ preferred landing positions (PLPs) have been shown to fall to the left of words’ centers when reading English^25^ or to the right of words’ centers for right-to-left scripts.^26^ Slower readers or ones with reading difficulties often perform shorter saccades, longer fixations, and more fixations per word,^27^^,^^28^^,^^29^^,^^30^ attending to local cues (smaller sequences of letters). It is therefore an interesting question whether under scotopic conditions, dealing with the foveal scotoma, readers will execute more fixations per word or shift their gaze away from photopic PLPs to locate word centers in the periphery, where acuity is better.

When recognizing faces, subjects usually direct their first fixation to the face’s eyes area,^31^^,^^32^^,^^33^^,^^34^ and this PLP is associated with improved performance.^35^^,^^36^^,^^37^ The face inversion effect, or the reduction in recognition performance for inverted compared to upright faces, has also been extensively studied.^38^^,^^39^^,^^40^ This effect has been used as evidence that unlike other object categories, upright faces are processed holistically,^41^ while inverted ones are processed more locally.^42^ Face inversion leads participants to make more fixations per face, more fixations to the lower parts of the face (mouth and chin), and proportionally fewer fixations to the upper parts of the face relative to upright faces.^43^^,^^44^ Therefore, if scotopic conditions harm the holistic nature of face recognition, we would expect participants to make more fixations to the lower parts of an upright face and exhibit no inversion effect.

Potential differences between scotopic and photopic conditions in performance or in eye-movement behavior might result from the degraded acuity of the rod system, rather than factors specific to rod vision (such as dark conditions, the rod scotoma, etc.). To that end, we also tested participants under photopic conditions while degrading their acuity to resemble that attained under scotopic conditions.

## Results

### Acuity

To test acuity, participants were asked to identify the orientation of a letter E that appeared on both sides of a fixation point and changed size according to a staircase procedure. The acuity threshold was measured at varying eccentricities under photopic, blurred, and scotopic conditions.

**Figure 1:**
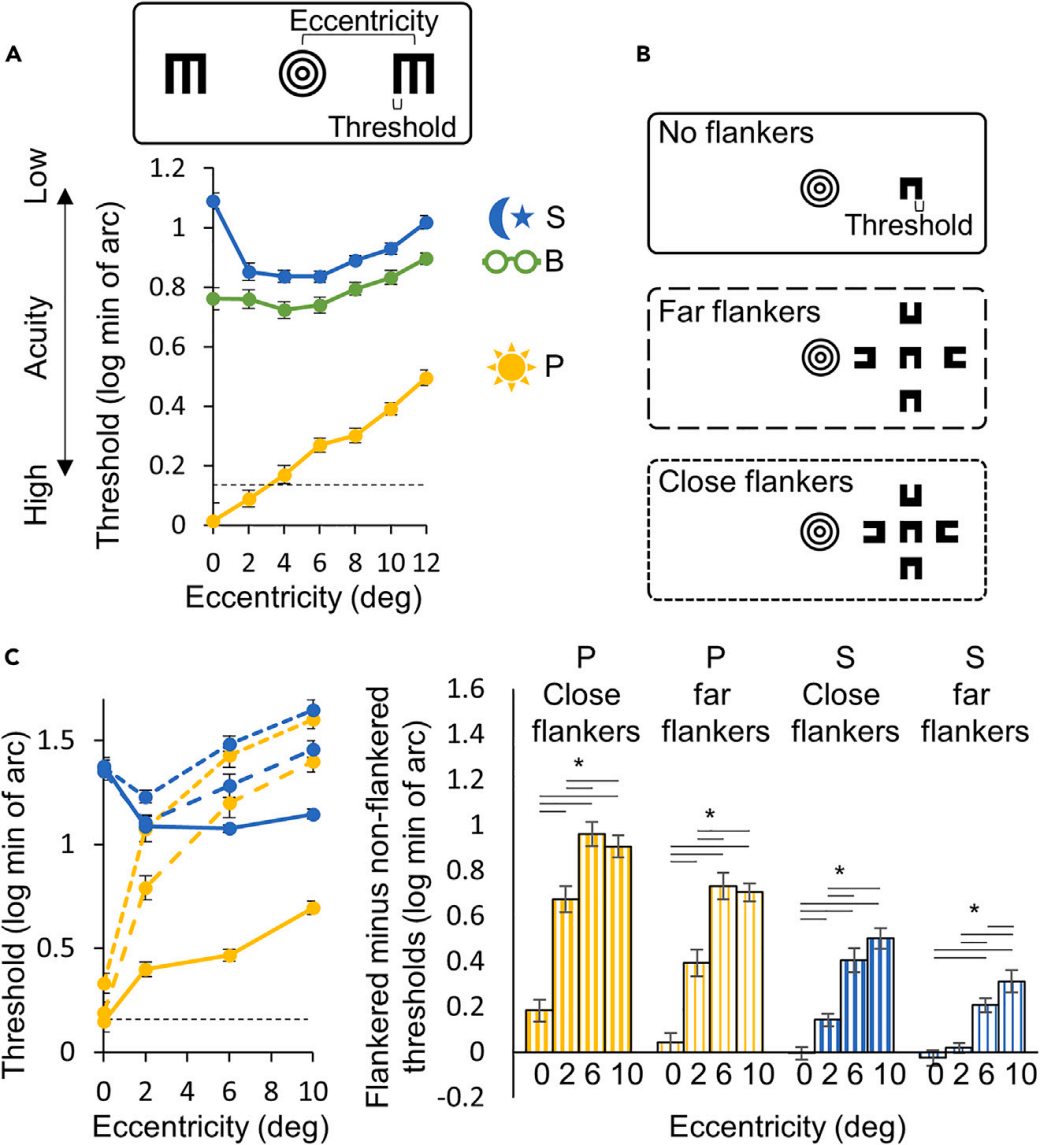
Acuity and Crowding screenshots and results (A) On the top, a screenshot showing the two E letters presented at a certain eccentricity and the fixation point in the center of the screen. The thickness of the E letter leg (which is a fifth of its width/height) averaged across the last six reversals was defined as the acuity threshold for each condition and eccentricity. On the bottom, the log of the acuity thresholds in the three conditions (photopic - yellow; blurred - green; scotopic - blue) as a function of eccentricity. Zero log threshold corresponds to 20/20 vision. (B) Screenshots showing the C target with no flankers, far flankers, and close flankers presented at a certain eccentricity, with the fixation point in the center of the screen. The thickness of the C target leg (which is a third of its width/height) averaged across the last six reversals was defined as the acuity threshold for each condition, eccentricity, and flanker situation. (C) On the left, the log of the acuity thresholds in photopic and scotopic conditions as a function of eccentricity. Flanker situation is represented by the line integrity (no flankers - solid; far flankers - long dashed; close flankers - short dashed). On the right, the difference between flanked and non-flanked acuity for close (thick line filling) and far (thin line filling) flankers under photopic and scotopic conditions for each eccentricity. Horizontal lines under the asterisk represent a significant (p < 0.05) difference in a t test performed between two eccentricities. Dashed lines in graphs represent the minimal threshold that could be measured directly. Measurements below these lines are extrapolations (see STAR methods for more details). Data are represented as mean ± SEM.

The acuity threshold pattern along the eccentricity axis differed between all conditions (see interaction terms in Table S1 ANOVA1, 2a, 2b, 2c). Under photopic conditions, participants demonstrated the highest acuity in the center of the visual field, with a reduction at larger eccentricities (Figure 1A). Under scotopic conditions, participants' acuity was at its worst in the center and at best 2–6° from the center.

Under scotopic conditions, acuity was significantly lower than under photopic conditions (Table S1 ANOVA1, 2b) across all eccentricities (Table S2.1). Under blurred conditions, acuity was also significantly lower than under photopic conditions (Table S1 ANOVA1, 2a) across all eccentricities (Table S2.2), but still higher than under scotopic conditions (Table S1 ANOVA1, 2c) at eccentricities 0, 8, 10, and 12 (Table S2.3).

### Crowding

Similar to the acuity experiment, participants’ acuity threshold at varying eccentricities was measured using a C orientation recognition task with/without close/far flankers (Figure 1B) under photopic and scotopic conditions.

Figure 1C depicts the crowding experiment results. Conditions, flankers, and eccentricity significantly influenced the acuity threshold (Table S1 ANOVA3). Moreover, under both conditions, the crowding effect increased with eccentricity: the difference between flanked and non-flanked thresholds was generally smallest at 0, larger at 2, and largest at 6 and 10° in both scotopic and photopic conditions, for both close and far flankers (Figure 1C; right; Tables S2.11–S2.14).

The crowding experiment was designed to assess the crowded acuities under scotopic and under photopic conditions *separately*, as well as how each of them changes along the eccentricity axis. In Figure 1C, the crowding effect seems larger for the photopic than for the scotopic condition. However, this observation might be misleading, as it could be explained by the high acuity threshold in the non-flanked scotopic condition. For a discussion regarding a direct comparison between lighting conditions, see Figure S2.

### Reading

To test reading performance and eye movement patterns, participants read 10 consecutive sentences in a learning phase that they were then asked to identify among 20 consecutive sentences in a test phase. Besides the success rate, which was obviously determined by the test phase, all results were based on the learning phase, since participants were more likely to read the whole sentence in this section.

**Figure 2:**
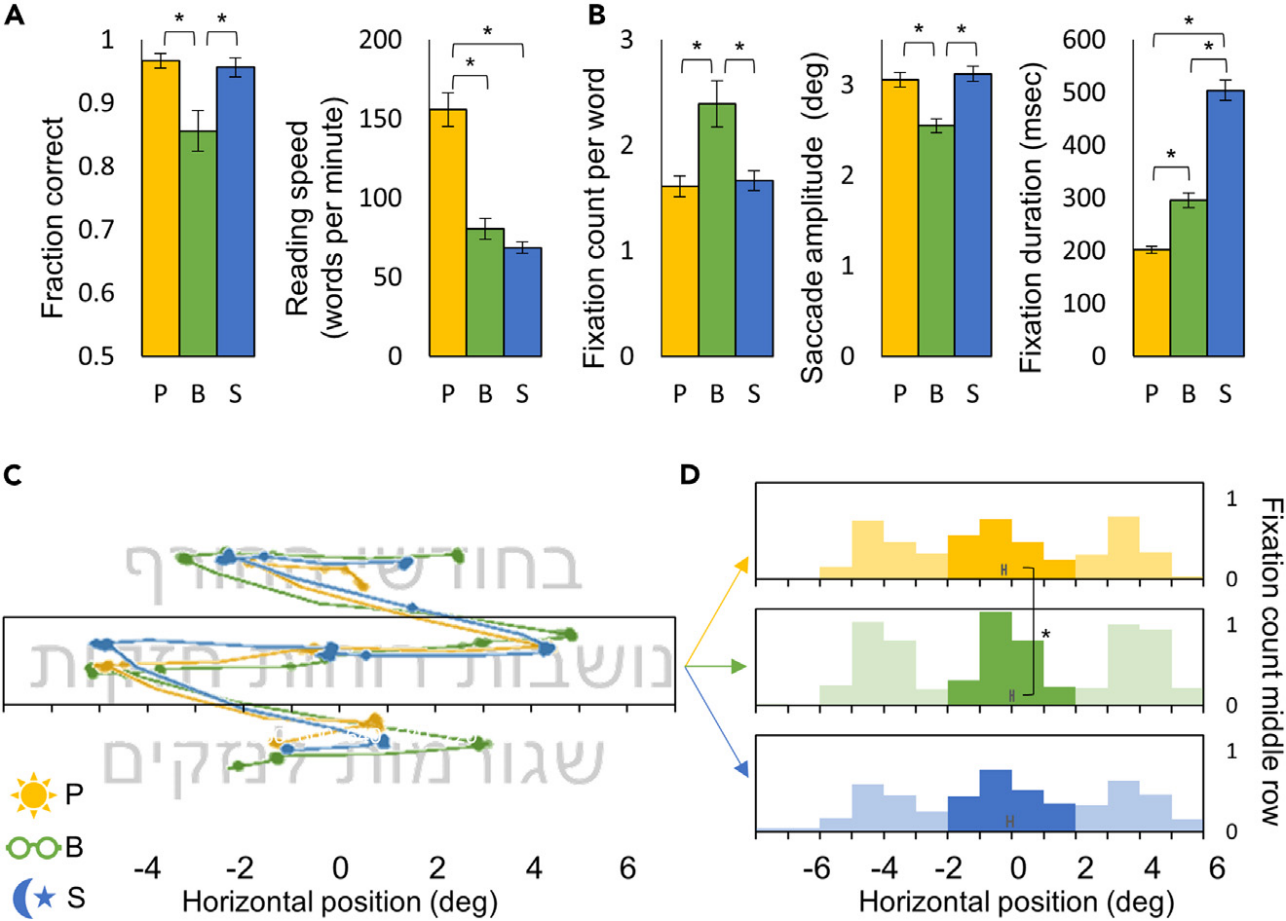
Reading performance and eye tracking results (A) On the left, the fraction of correct responses under photopic (yellow), blurred (green), and scotopic (blue) conditions. On the right, the reading speed under each condition. (B) The number of fixations per word (left), the saccade amplitude (center), and the fixation duration (right) under each condition. (C) Three eye tracking traces from one participant taken from three trials during the learning phase, each trace from a different condition. The image behind the traces shows a typical 7-word sentence and the location and length of the 4th word, which was equalized between trials. (D) Histograms of the different conditions showing the number of fixations in each horizontal 1-degree eccentricity bin for the middle row of the sentence (framed with a rectangle in C). The darker color represents the middle word location, where fixation horizontal locations were averaged (symbols inside the histograms) and compared between conditions. Data are represented as mean ± SEM. Asterisks represent a significant (p < 0.05) difference in a t test.

Subjects performed the task almost flawlessly under both scotopic and photopic conditions and less so under the blurred condition (Figure 2A left; Table S2.4). The high success rate is probably a result of the large letter size of around 50 min of arc, demanding roughly 10 min of arc threshold to distinguish between letters. This corresponds to 1 log min of arc threshold, which, as can be seen in Figure 1C and is about the minimal font size that can be seen across all crowded conditions. Nevertheless, reading speed was much slower under scotopic and blurred conditions than under the photopic condition (Figure 2A right; Table S2.5).

Eye tracking results show that while reading speed was slower under the blurred condition due to more fixations per word, shorter saccades, and longer fixation durations, under the scotopic condition only the latter caused the slow reading speed (Figure 2B; Tables S2.6–8).

Figure 2C shows eye tracking traces (representing the presumed positions of the fovea) of a typical participant reading a sentence under photopic, blurred, and scotopic conditions. The figure demonstrates the additional fixations and shorter saccades under the blurred condition while showing the similarity in fixation positions between conditions. Under all conditions, most fixations lay slightly to the left of a word center (Figure 2D; histograms). Analysis of the average horizontal eye position on the word in the middle of the screen revealed that under the blurred condition, fixations were slightly further to the right than under the photopic condition (Figure 2D; symbols; Table S2.9). However, no differences in fixation position, horizontal or vertical, were found between photopic and scotopic conditions (Tables S2.9–10).

### Face matching

To test for face recognition, participants were asked whether two faces shown on both sides of the screen shared the same identity or not. The left faces in all pairs were facing forward and aligned to the same location, and they were therefore used for eye tracking analysis. The test was administered with blocks of upright and inverted faces under photopic, blurred, and scotopic conditions.

**Figure 3:**
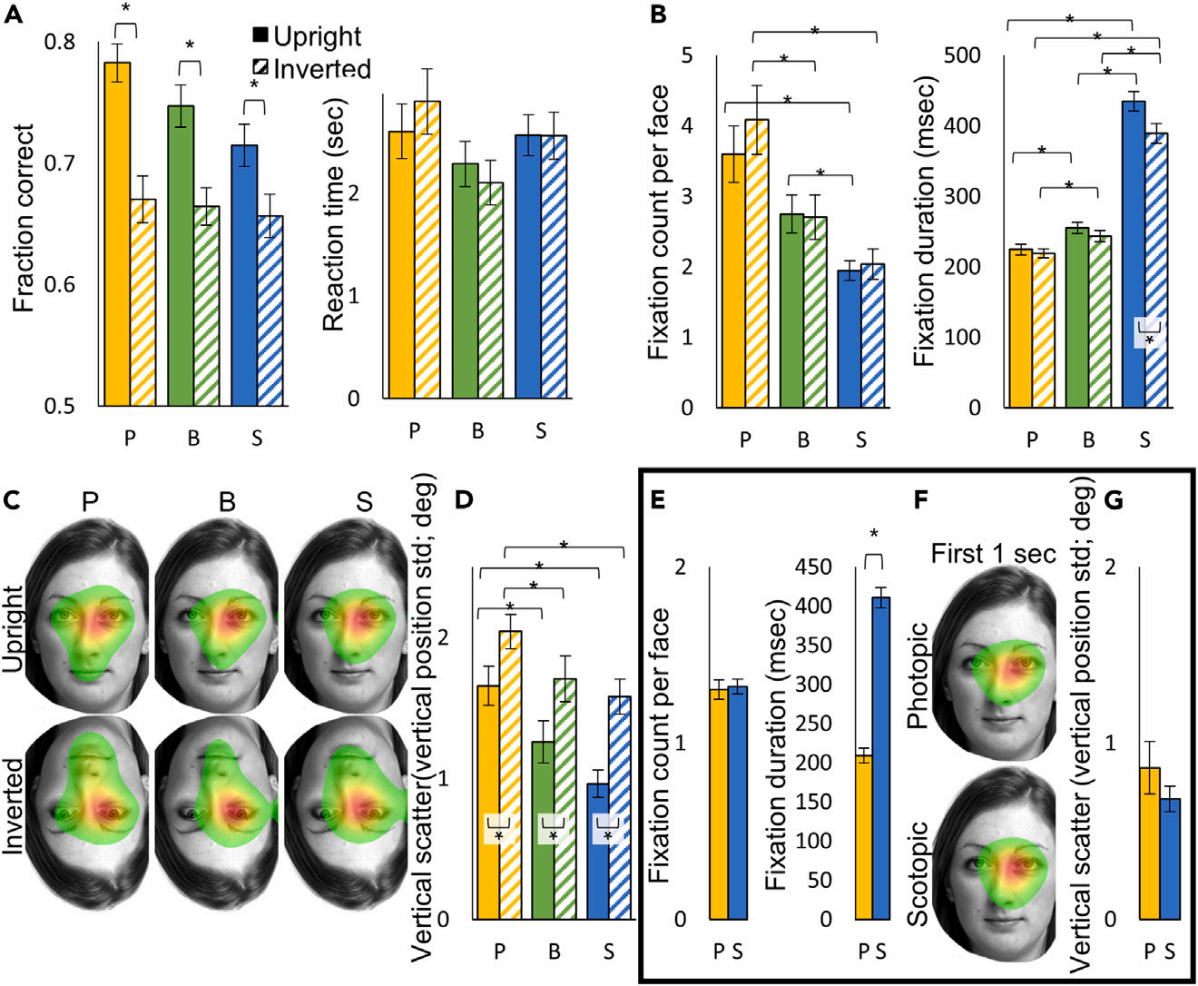
Face Matching performance and eye tracking results (A) On the left, the fraction of correct responses under photopic (yellow), blurred (green), and scotopic (blue) conditions and under both orientations (striped - inverted). On the right, the reaction time under each condition and orientation. (B) The number of fixations per face (left) and the fixation duration (right) under each condition and orientation for the left face. (C) Six fixation maps averaged across participants for each condition and orientation showing the fixated areas on the left face (normalized from green - fixated at 10% of the time, to red - most fixated). (D) Vertical scatter of the fixations on the left face under each condition and orientation, represented by averaging the vertical position standard deviation across participants. In the frame: (E–G) Eye tracking results for the left face in upright face pairs during the first 1s in the photopic and the first 2s in the scotopic condition. (E) The number of fixations per face (left) and the fixation duration (right) under each condition. (F) Fixation maps averaged across participants under each condition showing the fixated areas on the left face (normalized from green - fixated at 10% of the time, to red - most fixated). (G) The vertical scatter of the fixations, calculated as in (D). Data are represented as mean ± SEM. Asterisks represent a significant (p < 0.05) difference in a t test.

Participants performed above chance in all conditions and face orientations (Figure 3A; left). Performance was significantly affected by orientation (Table S1 ANOVA4), and this inversion effect was observed under all conditions (Table S2.15). No effect of either condition or orientation was observed on reaction time (RT, Figure 3A; right; Table S1 ANOVA5).

Eye tracking analysis of the left face showed that even though RT was similar between conditions, participants executed different numbers of fixations under the different conditions (Figure 3B; left; Table S1 ANOVA6), with significantly more fixations under the photopic than the scotopic condition when viewing both upright and inverted faces (Tables S2.16–18). This effect complements the significantly different fixation durations under the different conditions (Figure 3B; right; Table S1 ANOVA7), where similarly to the reading task, fixation duration was longest under scotopic and shortest under photopic conditions when viewing both upright and inverted faces (Tables S2.19–22).

Fixation maps of the left face showed that under all conditions and orientations, most fixations were executed to a similar location around the eyes region (Figure 3C). Fixation horizontal (but not vertical) position depended on condition and orientation (ANOVA8-9), but no differences in fixation position were found between photopic and scotopic conditions (Tables S2.23–26). However, the area covered by the fixations decreased as the conditions progressed (from photopic to blurred to scotopic), while also increasing when faces were inverted. Both vertical and horizontal fixation scatter (represented by the standard deviation of the fixation positions) depended on condition and orientation (Figure 3E; Table S1 ANOVA10-11). Under scotopic conditions, there was less vertical scatter of fixation positions than under photopic conditions for both upright and inverted faces (Figure 3D; Tables S2.27–34).

To summarize, under scotopic conditions, participants made fewer, longer, and less scattered fixations than under photopic conditions.

To better understand whether the larger vertical scatter under photopic conditions stemmed from a general scanning pattern throughout the trial or from additional later fixations executed under photopic conditions, we conducted an additional analysis of the upright face recognition data. In the new analysis, we examined only the first fixations, equalizing their number by comparing the first 1 s of the photopic trials with the first 2 s of the scotopic trials (since fixation duration was roughly doubled under scotopic compared to photopic conditions). Indeed, under these restrictions, the numbers of fixations per face equalized (Figure 3E; left; Table S2.35), while fixation duration was still higher under the scotopic than the photopic condition (Figure 3A; right; Table S2.36). More importantly, the difference between scotopic and photopic conditions in vertical scatter disappeared (Figures 3F and 3G; Table S2.37), and there were no other differences in fixation position/scatter parameters (Tables S2.38–40). Similar results were obtained when we repeated the face matching experiment while presenting the stimulus pairs for a restricted 1.25 s in the photopic condition and 2.5 s in the scotopic condition (Figure S1). Together, our results suggest that the initial fixations were executed to similar locations under both scotopic and photopic conditions.

## Discussion

Overall, we found that scotopic crowding increases with eccentricity and that high-level visual tasks are solved in a similar manner under scotopic as under photopic conditions. Under scotopic conditions, participants continue to read accurately, recognize faces, and show a face inversion effect. While performing these tasks, they continue to execute fixations to similar locations as under photopic conditions, despite our finding that crowded acuity under scotopic conditions is best 2° into the periphery. The only consistent difference we observed between lighting conditions was the longer fixation duration under scotopic conditions, doubled in length compared to photopic conditions.

Simunovic and Calver^45^ tested flanking bars interference on Landolt C target identification under scotopic conditions (only at 10° degrees into the periphery) and found a smaller interference effect than expected from photopic crowding. In a similar task, when directly comparing between lighting levels, Musilová et al.^46^ did not find consistent luminance-induced change in the interference magnitude. Our crowding results, which were obtained using flanking letters rather than bars, did not show reduced interference under scotopic conditions. The difference between Simunovic and Calver’s and our interference magnitudes is not surprising. Flanking bars have been shown to have a smaller interference effect on identification than flanking letters.^47^ It has been argued that while the first configuration represents contour interaction, the latter measures crowding.^47^

As mentioned in the introduction, it could take days,^18^ months, or even years^19^ for subjects to develop spontaneous fixations patterns to utilize peripheral retinal locations following central vision loss. Consequently, it is very plausible that even if averted vision could help recognition under scotopic conditions, eye movements would still be automatically performed as under photopic conditions. Nevertheless, we found no deficit in high-level vision caused by this retained behavior. In the following sections, we suggest that this intact performance relies on unharmed global perception.

### Reading

It has been shown previously that scotopic reading is slower than photopic reading and that words flashed around the center of the visual field (0–5°) are easier to read than words flashed in the periphery.^9^ While both scotopic and blurred conditions showed reduced reading speed in the current study, only under the blurred condition were there lower accuracy, more fixations, and shorter saccades, which suggest the use of more local cues. These results imply that while slow reading under the blurred condition probably reflects a reading difficulty, slow reading under the scotopic condition probably reflects the inherent fixation duration elongation witnessed in all tasks, not a decline in reading expertise or a shift to more local word perception.

### Face recognition

When given unlimited time to explore the face, participants performed fewer fixations to the nose and mouth region as acuity decreased: most under photopic, less so under blurred, and least under scotopic conditions. High acuity might allow comparison of local features, such as freckles or beauty marks, outside the eyes region. Therefore, it is reasonable that we found fewer additional fixations as acuity decreased, as low acuity prevented these features from being processed. We suggest that face recognition under scotopic conditions is performed in a similar manner as under photopic conditions, excluding the exploration for local features which are inaccessible under these conditions. Moreover, the similarity between scotopic and blurred condition results (with fewer fixations and less scatter, for instance) further strengthens the idea that global perception is involved: it has previously been shown that blurred photopic conditions decrease only the feature-based and not the global aspects of face recognition.^48^ Taking together these eye-tracking results along with the high recognition rate and significant inversion effect, we believe faces are processed globally under scotopic conditions.

### Averted vision might disrupt global perception

When reading under scotopic conditions, participants did not appear to shift their gaze away from the (photopic) PLPs. When recognizing faces under scotopic conditions, participants’ average eye position was similar to that under photopic conditions. While these eye movements might only result from an automatic behavior, there is cortical evidence as to why this behavior might be optimal for the use of global perception. In reading, shifting gaze away from a single letter might make sense under scotopic conditions, since it locates the letter in the periphery, where acuity is best. However, since word recognition is thought to rely on the external features or the word’s envelope,^24^ shifting the gaze away from the naturally acquired PLP could be problematic, since it shifts the word’s envelope to a different retinal location. Consequently, this could misplace the word’s envelope outside the visual attention span^49^ and outside an acquired receptive field in the ventral occipito-temporal cortex reading circuitry,^50^ potentially interfering with word recognition. Similarly, the foveal location of the receptive fields in the ventral occipito-temporal cortex reading circuitry has been suggested as the reason that reading is much slower in the periphery, even when letters are appropriately scaled.^50^^,^^51^

For faces, it has been suggested that the first few fixations to the eyes area are enough to initiate face recognition.^35^ Shifting the gaze away from the eyes to locate them in a retinal area with better acuity under scotopic conditions might interfere with recognition due to receptive fields' spatial properties in face-selective cortical regions. Population receptive fields in these regions have been shown to cover visual space unevenly, centered slightly to the lower left of fixation, making it reasonable to fixate in the upper right area of the face (as between the right eye and the nose bridge). Averting the gaze might move the viewed face outside the specific receptive field coverage in face-selective regions. Furthermore, since population receptive fields in these areas are three times as large as those in V1,^52^ the foveal scotoma might not interfere due to the vast pooling in these regions. Consequently, fixating in similar positions under all conditions could potentially support optimal global perception.

To summarize, spatially, it has been suggested that people can act in two different attention modes—global and local.^53^ For both reading^54^ and face recognition, global perception is considered more efficient and representative of perceptual expertise. We suggest that our results reflect dominant global perception under scotopic conditions.

### Temporal delay

The most pronounced difference we observed, regardless of task, was that the average fixation duration under scotopic conditions was doubled in length compared to photopic conditions. A similar difference of 200 ms was also observed when participants performed a visual search task under photopic and scotopic conditions.^17^ Some of this difference can be attributed to retinal neural sources: the VEP (visual evoked potential) latency delay has been shown to increase with decreasing stimulus intensity, resulting in a difference of up to 100 ms between scotopic and photopic conditions.^55^ Another contributing factor to this difference could be the saccade latency, which was also found to increase with decreasing stimulus intensity, resulting in a 100 ms difference between scotopic and photopic conditions.^56^ Furthermore, as suggested by Paulun et al.^17^ (2015), longer fixations might help produce longer temporal integration in the rod system in order to increase target detectability. Paulun et al. also offered a possibility that observers fixate longer under scotopic conditions to covertly shift their attention across a larger peripheral area surrounding the fovea. However, since we found that the periphery is susceptible to crowding, and since the fixation elongation seems implicit and identical across tasks, we find the first option more probable. If, indeed, longer stimulus durations are preferable under scotopic conditions due to longer temporal integration, performance reduction under these conditions should be discussed carefully. If a stimulus is time limited (for example, global form^10^), a deficit in recognizing this stimulus might not reflect an impairment in the specific tested visual function, but rather a general visual system requirement for longer presentation durations.

### Conclusion

Despite low-level limitations, high-level visual tasks, even those that rely on foveal input, are solved in a similar manner under scotopic as under photopic conditions. We might read slower, but we read accurately and move our eyes as skilled readers. When recognizing faces, we remain well above chance level while still using global perception. Overall, we do not change our visual strategies to accommodate to dark conditions, but instead we retain the same global visual behaviors we use in the light. Receptive fields’ spatial properties might explain why similar eye-movement behavior complements high-level global perception.

### Limitations of the study

The fact that this study tests only normally sighted individuals, whose visual system developed using cone input, might potentially impact our conclusions. The similar behavior under photopic and scotopic conditions could arise from optimal behavior common to both cone and rod input. Alternatively, this behavior may have been learned as the visual system developed under cone input and is now automatically executed under rod input as well. To distinguish between these possibilities, it is insufficient to study only the sighted population, and testing congenital rod monochromats should be considered.

## STAR★Methods

### Key resources table

**Table:** 

REAGENT or RESOURCE	SOURCE	IDENTIFIER
**Software and algorithms**		

ExperimentBuilder	https://www.sr-research.com/software/	N/A
DataViewer	https://www.sr-research.com/software/	N/A

### Resource availability

#### Lead contact

Further information and requests for resources should be directed to and will be fulfilled by the lead contact, Netta Levin (netta@hadassah.org.il).

#### Materials availability

This study did not generate new unique materials.

#### Data and code availability

(1) data reported in this paper will be shared by the lead contact upon request.
(2) This paper does not report original code.
(3) Any additional information required to reanalyze the data reported in this paper is available from the lead contact upon request.

### Experimental model and study participant details

24 adults (11 females; age: 26.5 ± 8.0 Years; Caucasians) participated in the acuity, reading, and face matching experiments. Half of them (N = 12; 3 females; age: 27.0 ± 9.7 Years) also participated in the crowding experiment and the timed face matching experiment. All subjects had normal vision without the use of corrective lenses, spoke and read Hebrew as their first language, and reported no known problems with face recognition, reading, or night vision. Data from one subject were discarded in the acuity experiment since she achieved a floor effect at all eccentricities under scotopic conditions, despite performing almost flawlessly in reading and above chance in face matching under these conditions. Data from a different subject were discarded in the face matching experiment due to a technical error. All participants gave written informed consent, and the study was approved by the ethical committee of the Hadassah Hebrew University Medical Center.

### Method details

#### Experimental procedure

Acuity, reading, and face matching experiments were conducted under photopic, scotopic and blurred (photopic) conditions. Crowding and timed face matching experiments were conducted later on (on the subset of the participants who agreed to return for these follow-up experiments), and they were only conducted under photopic and scotopic conditions due to the authors’ prioritization. Experiments under photopic and blurred conditions were performed in a lit room, with blurring achieved using +5.00 D prescription glasses. Experiments under scotopic conditions were performed in a dark room with a screen covered by neutral density filters (see below), and they were preceded by 20 min of dark adaptation. All experiments were designed using Experiment Builder software (SR-Research). During the experiments, participants used a chin and head rest to prevent head motion, while an EyeLink 1000 eye tracker (SR-research) was used to collect monocular eye tracking data at 500 Hz.

The order of lighting conditions and the specific stimuli included in each lighting condition (faces and sentences) were counterbalanced across participants and conditions. However, all subjects began their session with the acuity experiment under photopic conditions, as this was used to familiarize subjects with the setup and screen.

#### Screen

Stimuli were presented on a 1280 × 1024 pixel (37.5 × 30 cm) screen. Subjects sat 65 cm away from the screen, such that 39.8 pixels corresponded to 1 degree of visual angle. For experiments under photopic and blurred conditions, the screen had luminance of 144 cd/mˆ2 for white and 0.15 cd/mˆ2 for black, measured using a Mavo-Monitor USB (Gossen) photometer. For experiments under scotopic conditions, a screen of the same size, luminance, and resolution was used. 3 identical neutral density filters were added, each reducing the screen’s luminance by a factor of 40 according to our photometer measurements. According to this calculation, the resulting screen luminance under scotopic conditions was ∼0.002 cd/mˆ2 for white and ∼ 2E-06 cd/mˆ2 for black (extrapolated).

#### Fixation point

In all experiments, the background was white, and the fixation point was constructed from a set of black concentric circles (Figure 1A) that covered 3.75°. In this manner, the fixation point was visible under all conditions, and when present, participants were instructed to fixate in its center.

#### Calibration

Each experiment began with an EyeLink 1000 Plus built-in 5-point calibration procedure. The built-in fixation point was replaced by our custom fixation point (described above). Participants were instructed to fixate in the middle of the concentric circles. Under scotopic conditions, when the screen was projecting only low-luminance stimuli, fixating on this fixation point meant that the inner circles were not visible. In every experiment, a new trial was initiated only after fixation was detected within the fixation point (see below for more details). In this manner, if, for some reason (drift, head motion), calibration was no longer valid, the experiment stopped, and the experimenter recalibrated the eye positions before continuing.

#### Acuity

A trial began when participants successfully fixated on a central fixation point (within 1.25° radius from the fixation point center, monitored by the eye tracker). After 200ms, a black letter E (with leg width a fifth of its height and width) was presented for 200 ms at one of four orientations (Figure 1A). If participants did not maintain fixation during stimulus presentation (within 1.25° radius), the trial was aborted and recycled. Subjects indicated the orientation of the E; they were also allowed to respond that they did not know the orientation, which was counted as an incorrect response.

Stimulus size (both letter size and leg width) was adjusted using a 1-down 1-up staircase procedure, with possible levels of 1.5, 3, 4.5, 6, 9, 13.5, 19.5, 28.5, 39, and 64.5 min of arc (for the width of one leg of the E). The staircase terminated after 12 reversals, with the average stimulus size across the last 6 reversals counted as the final acuity threshold. In the case of a ceiling effect, i.e., a participant answering the 1.5 min of arc (1 pixel) stimulus correctly, a simulated reversal located on an incorrect trial for a 0.75 min of arc stimulus was added. This procedure served both to terminate the experiment even under a ceiling effect and to extrapolate the threshold to a value under 1.5 min of arc.

The staircase procedure was performed separately at eccentricities of 0, 2, 4, 6, 8, 10, and 12°. For eccentricities greater than 0, two stimuli with the same orientation were presented in each trial, one on each side of fixation. This was designed to make it easier for subjects to maintain central fixation. If the E stimulus overlapped with the fixation point (at 0 and 2° eccentricities), a white disk slightly larger than the E stimulus served as the stimulus background so that the fixation point would not interfere with stimulus recognition. Otherwise, the whole fixation point was present during the whole trial.

#### Crowding

The crowding experiment was structured similarly to the acuity experiment. A trial began when participants successfully fixated on a central fixation point (within 1.25° radius from the fixation point center, monitored by the eye tracker). After 200ms, a black squared C target (with leg width a third of its height and width) was presented for 200 ms at one of four orientations (Figure 1B). The target was presented either alone, with far flankers (with C height space in between), or with close flankers (with C leg width x 2 space in between). Flankers were identical C letters facing outwards from the C target. If participants did not maintain fixation during stimulus presentation (within 1.25° radius), the trial was aborted and recycled. Subjects indicated the orientation of the C target; they were also allowed to respond that they did not know the orientation, which was counted as an incorrect response.

For each block (no flankers, far flankers, close flankers), target size (both letter size and leg width) was adjusted using a 1-down 2-up staircase procedure, with possible levels of 1.5, 3, 4.5, 6, 9, 13.5, 19.5, 28.5, 39, and 64.5 min of arc (for the width of one leg of the C target). The staircase terminated after 8 reversals, with the average stimulus size across the last 6 reversals counted as the final threshold. In the case of a ceiling effect, i.e., a participant answering the 1.5 min of arc (1 pixel) stimulus correctly twice, a simulated reversal located on an incorrect trial for a 0.75 min of arc stimulus was added. This procedure served both to terminate the experiment even under a ceiling effect and to extrapolate the threshold to a value under 1.5 min of arc.

The staircase procedure was performed separately with the target at eccentricities of 0, 2, 6, and 10° to the right of the fixation point.

#### Reading

The reading experiment consisted of two phases: a learning phase and a test phase. Each trial began when participants fixated for 300ms on a fixation point (within 1.5° radius from the fixation point center, monitored by the eye tracker) in the upper region of the screen above the area where the sentence was due to appear. After pressing the space bar, the fixation point disappeared, and a 7-word Hebrew sentence was presented in 3 lines (with two, three, and two words in each line; Figure 2A). The sentence was presented in black Arial font, with an average letter size of ⅞ degree and a spacing between adjacent letters of ¼ degree. The fourth word was of similar length for all sentences, and its middle was always fixed to the center of the screen. In the learning phase, subjects were presented with 10 sentences (+one example) one at a time, and they were directed to read each sentence before pressing a key to move to the next one. In the test phase, subjects were presented with 20 sentences (+two examples) one at a time, and they were directed to press a key indicating whether each sentence was previously shown in the learning phase or not. Ten of the sentences (and one example) were shown previously, while the remainder were not. Responses were not subject to a time limit.

#### Face matching

All trials began when participants fixated for 200ms on a central fixation point (within 1° radius from the fixation point center, monitored by the eye tracker) and pressed the space key. Afterward, the fixation point disappeared, and subjects were presented with pairs of grayscale face images to the left and to the right of the previous fixation point (unlimited in time). Subjects responded whether the two faces in the pair showed the same identity or not.

Face pair stimuli were taken from the Glasgow Face Matching Task 2 dataset.^57^ On the left side of each pair, a forward-facing, high-quality face image was presented (Figure 3C), spanning about 12 × 9°. On the right side, a face of the same or different identity was presented, subject to alterations in head angle, pose, expression, or resolution. In all conditions, images were converted to grayscale and increased in contrast to 100% to make them easier to see in the dark, and the left face was adjusted such that its eyes appeared at the same position across images. To test for a face inversion effect, inverted face stimuli were generated by flipping the face pair images along the vertical axis.

In a given condition, subjects were shown a set of 50 upright and 50 inverted face pairs, with the order of upright and inverted blocks and the specific face pairs in each block counterbalanced across subjects. Face pairs were chosen such that each block had the same difficulty level (according to White et al.’s^57^ difficulty rating for each pair), and half of the pairs shared the same identity. For a given subject, each face pair appeared only once across all experimental sessions.

#### Timed face matching

For a subset of 12 subjects (3 females; age: 27.0 ± 9.7 Years), the face matching experiment was repeated after 137.5 ± 56.7 days using a variation of the task with time-restricted stimuli. Face pairs were shown for 2.5 s under scotopic conditions and for 1.25 s under photopic conditions, after which subjects made an identity judgment. Face pairs that were presented upright to a particular subject in the initial face matching experiment were inverted in the timed experiment, and vice versa (excluding one session where orientations were not reversed). For 7 of the 12 subjects, face pairs that were previously presented under photopic conditions were presented under scotopic conditions, and vice versa. For the remaining 5 subjects, face pairs were presented under the same lighting conditions as in the main experiment.

### Quantification and statistical analysis

Data Viewer software (SR-research) was used to define fixations and saccades using the program default parameters.

All statistical analyses were performed using R and RStudio software (RStudio Team (2020). RStudio: Integrated Development for R. RStudio, PBC, Boston, MA URL http://www.rstudio.com/). When there were more factors than the condition factor (eccentricity in the acuity experiment, eccentricity and flankers in the crowding experiment, and orientation in the face matching experiment), we conducted multi-factorial repeated-measures ANOVA. We reported degrees of freedom and p values for each factor or interaction in each ANOVA, applying Greenhouse-Geisser correction if Mauchly’s test of sphericity was significant (p < 0.05). When a factor proved to be significant, we followed the statistical analysis with the appropriate post-hoc paired t tests. When lighting condition was the only factor, only paired t tests were performed. In all t tests, we corrected the p value using Bonferroni correction for multiple comparisons.

All variable averages, standard error of the means and t tests’ p values (when smaller than 0.05) are shown in the graphs, and all statistical analysis results are presented in the Tables S1 and S2.
