# Supplementary material for: Seeing in the dark: High-order visual functions under scotopic conditions
Source: iScience. 2024 Jan 17;27(2):108929. doi: 10.1016/j.isci.2024.108929 (PMC10844829; doi:10.1016/j.isci.2024.108929)
Supplement: Document S1. Figures S1 and S2 and Tables S1 and S2 [file mmc1.pdf]

iScience, Volume *27*

Supplemental information

Seeing in the dark: High-order visual functions under scotopic conditions

Ayelet McKyton, Deena Elul, and Netta Levin

ANOVA	Experiment	Dependent variable	Factor 1 X Factor 2 (X Factor 3)	Factor 1 main effect		Factor 2 main effect		Interaction 1-2	
ANOVA1	Acuity	acuity threshold	7 eccentricities X 3 conditions	F(6,132)= 36.09	p < **0.0001**	F(2,44)= 523.85	p < **0.0001**	F(12,264)= 18.62	p < **0.0001**
ANOVA2a	Acuity	acuity threshold	7 eccentricities X 2 conditions (photopic/blur)	F(6,132)= 32.03	p < **0.0001**	F(1,22)= 466.51	p < **0.0001**	F(6,132)= 13.73	p < **0.0001**
ANOVA2b	Acuity	acuity threshold	7 eccentricities X 2 conditions (photopic/scotopic)	F(6,132)= 30.41	p < **0.0001**	F(1,22)= 1770.82	p < **0.0001**	F(6,132)= 29.68	p < **0.0001**
ANOVA2c	Acuity	acuity threshold	7 eccentricities X 2 conditions (scotopic/blur)	F(6,132)= 27.39	p < **0.0001**	F(1,22)= 31.59	p < **0.0001**	F(6,132)= 38.64	p < **0.0001**
ANOVA3	Crowding	acuity threshold	4 eccentricities X 3 flankers X 2 conditions	F(3,33)= 157.59	p < **0.0001**	F(2,22)= 246.66	p < **0.0001**	F(6,66)= 47.86	p < **0.0001**
	**Factor 3 main effect:** F(1,11)= 417.67; p < 0.0001 **Interaction 2-3:** F(2,22)=109.67; p < 0.0001 **Interaction 1-3:** F(3,33)=163.73; p < 0.0001 **Interaction 3 way:** F(6,66)=7.98; p < 0.0001								
ANOVA4	Face Matching	fraction correct	3 conditions X 2 orientations	F(2,44)= 2.68	p = 0.080	F(1,22)= 68.23	p < **0.0001**	F(2, 44)= 1.77	p = 0.182
ANOVA5	Face Matching	reaction time	3 conditions X 2 orientations	F(2,44)= 2.77	p = 0.074	F(1,22)= 0.11	p = 0.746	F(2,44)= 2.81	p = 0.071
ANOVA6	Face Matching	fixation count	3 conditions X 2 orientations	F(2,44)= 14.44	p < **0.0001**	F(1,22)= 1.32	p = 0.263	F(2,44)= 1.57	p = 0.220
ANOVA7	Face Matching	fixation duration	3 conditions X 2 orientations	F(2,44)= 292.76	p < **0.0001**	F(1,22)= 19.10	p = **0.0002**	F(2, 44)= 8.76	p = **0.0006**
ANOVA8	Face Matching	vertical position	3 conditions X 2 orientations	F(2,44)= 2.84	p = 0.069	F(1,22)= 1.97	p = 0.175	F(2,44)= 1.59	p = 0.215
ANOVA9	Face Matching	horizontal position	3 conditions X 2 orientations	F(2,44)= 6.32	p = **0.004**	F(1,22)= 6.79	p = **0.020**	F(2,44)= 0.05	p = 0.953
ANOVA10	Face Matching	vertical scatter	3 conditions X 2 orientations	F(2,44)= 13.77	p < **0.0001**	F(1,22)= 34.05	p < **0.0001**	F(2, 44)= 1.51	p = 0.233
ANOVA11	Face Matching	horizontal scatter	3 conditions X 2 orientations	F(2,44)= 3.68	p = **0.033**	F(1,22)= 20.64	p = **0.0002**	F(2,44)= 1.27	p = 0.291

Table S1. ANOVA statistical results. Degrees of freedom are presented for the original ANOVA tests, and the p-values are Greenhouse-Geisser corrected where appropriate.

T test	Experiment	Dependent variable	Comparisons	Multiple comparisons	T-test results	Corrected p values
2.1	Acuity	acuity threshold	P vs S	7 eccentricities: 0,2,4,6,8,10,12	t(22) =-15.63,-27.73,-25.29,-18.22,-21.57,-18.49,-18.4	**p<0.0001,p<0.0001,p<0.0001,p<0.0001,p<0.0001,p<0.0001,p<0.0001**
2.2	Acuity	acuity threshold	P vs B	7 eccentricities: 0,2,4,6,8,10,12	t(22) =-11.21,-19.16,-12.98,-14.12,-14.95,-16.65,-12.2	**p<0.0001,p<0.0001,p<0.0001,p<0.0001,p<0.0001,p<0.0001,p<0.0001**
2.3	Acuity	acuity threshold	S vs B	7 eccentricities: 0,2,4,6,8,10,12	t(22) =6.72,2.33,2.83,2.71,3.43,3.59,4.18	**p<0.0001**,p=0.203,p=0.068,p=0.088,**p=0.017,p=0.011,p=0.003**
2.4	Reading	fraction correct	P vs S, P vs B, S vs B	3 condition pairings	t(23) =0.61,3.78,2.97	p=1,**p=0.003,p=0.020**
2.5	Reading	reading speed	P vs S, P vs B, S vs B	3 condition pairings	t(23) =8.02,6.15,-2.15	**p<0.0001,p<0.0001**,p=0.125
2.6	Reading	fixation count	P vs S, P vs B, S vs B	3 condition pairings	t(23) =-0.46,-3.21,-3.54	p=1,**p=0.012,p=0.005**
2.7	Reading	saccade amplitude	P vs S, P vs B, S vs B	3 condition pairings	t(23) =-0.79,5.47,6.99	p=1.000,**p<0.0001,p<0.0001**
2.8	Reading	fixation duration	P vs S, P vs B, S vs B	3 condition pairings	t(23) =-15.47,-7.97,10.36	**p<0.0001,p<0.0001,p<0.0001**
2.9	Reading	horizontal position	P vs S, P vs B, S vs B	3 condition pairings	t(23) =-1.87,-3.27,-0.51	p=0.219,**p=0.010**,p=1
2.10	Reading	vertical position	P vs S, P vs B, S vs B	3 condition pairings	t(23) =0.04,-0.25,-0.35	p=1,p=1,p=1
2.11	Crowding	threshold difference	ecc: close flankers, P	0 vs 2, 0 vs 6, 0 vs 10, 2 vs 6, 2 vs 10, 6 vs	t(11) =-5.26,-10.37,-11.06,-7.32,-3.29,1.05	**p=0.002,p<0.0001,p<0.0001,p<0.0001,p=0.043**,p=1
2.12	Crowding	threshold difference	ecc: far flankers, P	0 vs 2, 0 vs 6, 0 vs 10, 2 vs 6, 2 vs 10, 6 vs	t(11) =-6.10,-9.24,-13.01,-6.47,-4.67, 0.39	**p=0.0005,p<0.0001,p<0.0001,p=0.0003,p=0.004**,p=1
2.13	Crowding	threshold difference	ecc: close flankers, S	0 vs 2, 0 vs 6, 0 vs 10, 2 vs 6, 2 vs 10, 6 vs	t(11) =-4.00,-6.58,-9.05,-4.89,-7.35,-3.00	**p=0.012,p=0.0002,p<0.0001,p=0.003,p<0.0001**,p=0.071
2.14	Crowding	threshold difference	ecc: far flankers, S	0 vs 2, 0 vs 6, 0 vs 10, 2 vs 6, 2 vs 10, 6 vs	t(11) =-1.16,-4.72,-4.72,-5.23,-5.68,-3.60	p=1,p=0.004,**p=0.004,p=0.002,p=0.0008,p=0.025**
2.15	Face Matching	fraction correct	upright vs inverted	photopic, blurred, scotopic	t(22) =6.32,3.80,3.11	**p<0.0001,p=0.003,p=0.015**
2.16	Face Matching	fixation count	P vs S	upright, inverted	t(22) =4.58,4.40	**p=0.0003,p=0.0005**
2.17	Face Matching	fixation count	P vs B	upright, inverted	t(22) =1.76,4.02	p=0.18,**p=0.001**
2.18	Face Matching	fixation count	S vs B	upright, inverted	t(22) =-2.84,-2.19	**p=0.0189**,p=0.077
2.19	Face Matching	fixation duration	P vs S	upright, inverted	t(22) =-20.62,-13.36	**p<0.0001,p<0.0001**
2.20	Face Matching	fixation duration	P vs B	upright, inverted	t(22) =-4.90,-3.56	**p=0.0001,p=0.003**
2.21	Face Matching	fixation duration	S vs B	upright, inverted	t(22) =17.10,12.91	**p<0.0001,p<0.0001**
2.22	Face Matching	fixation duration	upright vs inverted	photopic, blurred, scotopic	t(22) =0.97,1.997,4.457	p=1,p=0.175,**p=0.0006**
2.23	Face Matching	horizontal position	P vs S	upright, inverted	t(22) =-1.70,-1.15	p=0.206,p=0.519
2.24	Face Matching	horizontal position	P vs B	upright, inverted	t(22) =-3.39,-4.80	**p=0.005,p=0.0002**
2.25	Face Matching	horizontal position	S vs B	upright, inverted	t(22) =-1.48,-1.49	p=0.303,p=0.301
2.26	Face Matching	horizontal position	upright vs inverted	photopic, blurred, scotopic	t(22) =-1.92,-1.82,-1.62	p=0.201,p=0.245,p=0.354
2.27	Face Matching	vertical scatter	P vs S	upright, inverted	t(22) =4.66,4.44	**p=0.0002,p=0.0004**
2.28	Face Matching	vertical scatter	P vs B	upright, inverted	t(22) =2.89,2.97	**p=0.017,p=0.014**
2.29	Face Matching	vertical scatter	S vs B	upright, inverted	t(22) =-1.84,-1.12	p=0.159,p=0.544
2.30	Face Matching	vertical scatter	upright vs inverted	photopic, blurred, scotopic	t(22) =-3.00,-3.68,-6.61	**p=0.020,p=0.003,p<0.0001**
2.31	Face Matching	horizontal scatter	P vs S	upright, inverted	t(22) =2.20,0.97	p=0.076,p=0.679
2.32	Face Matching	horizontal scatter	P vs B	upright, inverted	t(22) =2.18,2.94	p=0.080,**p=0.015**
2.33	Face Matching	horizontal scatter	S vs B	upright, inverted	t(22) =-0.36,1.22	p=1,p=0.464
2.34	Face Matching	horizontal scatter	upright vs inverted	photopic, blurred, scotopic	t(22) =-2.65,-2.87,-2.91	**p=0.043, p=0.026,p=0.024**
2.35	Face Matching FF	fixation count	P vs S		t(22) =-0.36	p=0.720
2.36	Face Matching FF	fixation duration	P vs S		t(22) =-15.18	**p<0.0001**
2.37	Face Matching FF	vertical scatter	P vs S		t(22) =1.14	p=0.263
2.38	Face Matching FF	vertical position	P vs S		t(22) =0.57	p=0.569
2.39	Face Matching FF	horizontal scatter	P vs S		t(22) =-2.04	p=0.053
2.40	Face Matching FF	horizontal position	P vs S		t(22) =-0.96	p=0.343

Table S2. T-Test statistical results. P-values are Bonferroni corrected. FF - first fixations; P - photopic; S - scotopic; B - blurred; ecc - eccentricities.

Timed Face Matching experiment

To be further convinced that the differences in vertical scatter between photopic and scotopic conditions during face recognition stemmed only from additional later fixations in the photopic condition, we repeated the face matching experiment while presenting each stimulus for a restricted 1.25 seconds in the photopic condition and 2.5 seconds in the scotopic condition.

The fraction of correct responses was similar between conditions (Figure S1A; $t(11) = 0.94$; $p = 0.37$), with fewer fixations under photopic conditions (Figure S1B; left; $t(11) = -4.37$; $p = 0.001$). As observed in all our experiments, fixation durations were much longer under the scotopic than the photopic condition (Figure S1B; right; $t(11) = -13.03$; $p < 0.001$). As in the analysis of the first fixations in the original experiment, vertical scatter did not differ between conditions (Figure S1C&D; $t(11) = -0.54$; $p = 0.60$). These results suggest that initial fixations during face perception are executed similarly under scotopic and photopic conditions and allow at least some level of face recognition.

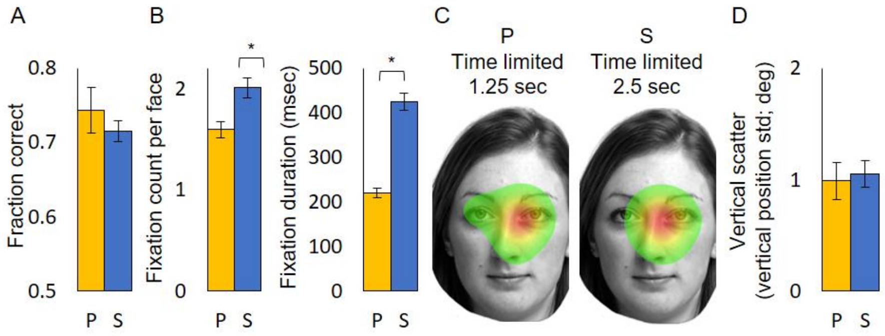

Figure S1. Timed Face Matching performance and eye tracking results.

(A) Timed face matching fraction of correct responses under the different conditions. (B) The number of fixations per face (left) and the fixation duration (right) under each condition. (C) Fixation maps averaged across participants under each condition showing the fixated areas on the left face (normalized from green - fixated at 10% of the time, to red - most fixated). (D) The vertical scatter of the fixations, represented by averaging the vertical position standard deviation across participants. Data are represented as mean ± SEM.

Photopic vs. Scotopic Crowding

Our crowding experimental procedure was designed to best imitate reading abilities, such that the target, the flankers, and the spacings all grew proportionally. As mentioned in the Results section, this design was not intended to directly compare between scotopic and photopic crowding. For that purpose, it would have been better to include more target-to-flanker spacings which are not proportional to the target size. However, even in this setting, we believe that the results do not suggest different mechanisms or major differences between scotopic and photopic crowding.

First, in Figure 1C, crowded scotopic and crowded photopic thresholds resembled each other at eccentricities of 6-10 degrees, both in the close and in the far flankers conditions. At eccentricities of 0-2 degrees, the observed differences between crowded photopic and scotopic thresholds are expected: since the uncrowded scotopic thresholds are higher than the crowded photopic thresholds, the crowded scotopic thresholds are limited to be at least as high as the uncrowded scotopic thresholds.

Second, in Figure 1C, one might observe the differences between crowded and uncrowded acuities in each condition and conclude that photopic crowding is greater than scotopic crowding. However, the small difference between the scotopic uncrowded and the scotopic crowded acuity could be attributed to the low acuity under scotopic conditions.

Third, in Figure S2, we analyze our data according to the spacing factor and compare them to Song et al.'[56] results (Figure S2A). Under photopic conditions (Figure S2B), our results resemble those of Song et al.: threshold increases with eccentricity and with flanker proximity. Under scotopic conditions (Figure S2C), similar results are obtained for eccentricities between 2-10 degrees. However, at 0 degrees, acuity is so compromised without flankers (infinite spacing factor) that it exceeds the expected crowding effect and masks it for small spacing factors.

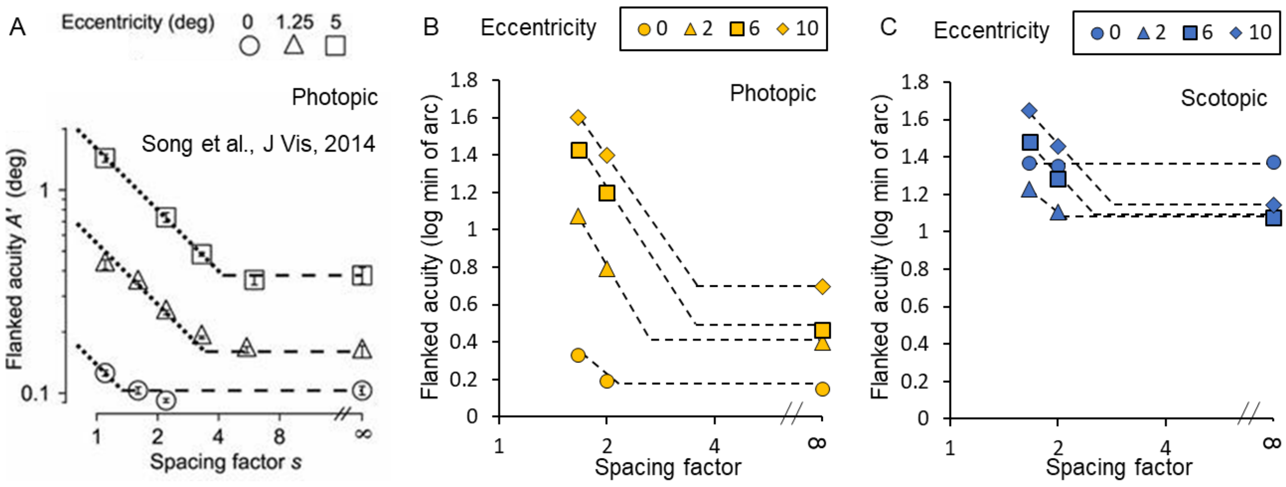

Figure S2. Comparison of the crowding results to Song et al.

(A) Flanked acuity taken from Song et al.[56] as a function of the spacing factor (target-flankers spacing as a multiple of the letter size), measured at eccentricities of 0, 1.25, and 5 degrees. (B+C) Our flanked acuity results as function of the spacing factor, measured at eccentricities of 0, 2, 6, and 10 degrees under photopic (B) and scotopic (C) conditions. Data are represented as mean.
